# The Value of Serum Midkine Level in Diagnosis of Hepatocellular Carcinoma

**DOI:** 10.1155/2015/146389

**Published:** 2015-02-08

**Authors:** Karim Y. A. Shaheen, Abeer I. Abdel-Mageed, Eslam Safwat, Ashraf M. AlBreedy

**Affiliations:** ^1^Department of Clinical and Chemical Pathology, Faculty of Medicine, Ain Shams University, Cairo 11566, Egypt; ^2^Internal Medicine Department, Faculty of Medicine, Ain Shams University, Cairo 11566, Egypt; ^3^Tropical Medicine Department, Faculty of Medicine, Ain Shams University, Cairo 11566, Egypt

## Abstract

*Background and Aim*. Identification of sensitive biomarkers to improve early diagnosis of HCC is needed. We aimed to evaluate serum midkine (MDK) as a biomarker for HCC diagnosis. *Patients and Methods*. 40 HCCs, 30 liver cirrhosis patients, and 30 healthy subjects were enrolled. Serum MDK using ELISA was measured in all included subjects. *Results*. Serum MDK was significantly elevated in HCC group compared to cirrhotic and healthy control groups (0.625 versus 0.15 and 0.125 ng/mL), respectively. No significant association was found between MDK and either BCLC stage, tumor diameter, tumor number, or AFP level. Receiver operating characteristic curve showed that best cutoff for MDK and AFP was 0.387 and 88.5 ng/mL, respectively. Area under the curve of MDK was significantly larger than that of AFP (0.941 versus 0.671). The sensitivity of MDK at 0.387 ng/mL for HCC diagnosis was significantly higher than that of AFP at cutoffs 20, 88.5, and 200 ng/mL (92.5 versus 62.5, 40, and 25%), respectively. Sensitivity of MDK reached 93.3% in patients with AFP <20 ng/mL. Moreover, MDK at 0.387 ng/mL had significant better sensitivity than AFP at 20 ng/mL in distinguishing HCC from BCLC 0/A (90 versus 40%). *Conclusion*. Serum MDK might be a potential diagnostic marker for HCC particularity in its early stages.

## 1. Introduction

Hepatocellular carcinoma (HCC) is the sixth most common cancer worldwide and the third most frequent cause of cancer-related death [1]. In Egypt, HCC represents 75% of malignant liver tumors. Liver cancer is the 5th most common cancer in both genders, the 6th in female representing 3.4% of cancers and 2nd in order in males after cancer urinary bladder representing 11.5% of all cancers. In 2010, liver cancer came in the 3rd order in both sexes (8.1%), 1st in males (12.1%) and 5th in females (4%) [2].

HCC is a condition which lends itself to surveillance as at-risk individuals can readily be identified because of the presence of underlying viral hepatitis or other liver diseases. The aim of surveillance is to obtain a reduction in disease-related mortality. This is usually achieved through an early diagnosis (stage migration) that, in turn, enhances the applicability and cost effectiveness of curative therapies [3].

The imaging test most widely used for surveillance is ultrasonography (US). Alpha-fetoprotein (AFP) is the most widely tested biomarker in HCC. However, AFP has a suboptimal performance as a serological test for surveillance for 2 reasons; firstly, fluctuating levels of AFP in patients with cirrhosis might reflect flares of HBV or HCV infection, exacerbation of underlying liver disease or HCC development [4]. Secondly, only a small proportion of tumors at an early stage (10–20%) present with abnormal AFP serum levels [5, 6]. In addition, other studies showed that des-*γ*-carboxyprothrombin [7] and AFP-L3 [8] were not superior to AFP for the diagnosis of early HCC. This highlights the need for new more reliable noninvasive recent biomarkers with better sensitivity and specificity for early diagnosis of HCC.

Midkine (MDK), also known as neurite growth-promoting factor 2 (NEGF2), is a basic heparin-binding growth factor of low molecular weight. In humans, it is encoded by the MDK gene on chromosome 11 [9]. It is a developmentally important retinoic acid-responsive gene product strongly induced during mid-gestation, hence the name midkine. Expression of the MDK gene in human adult tissues is extremely low and restricted. Mounting evidence has indicated that MDK plays a significant role in carcinogenesis-related activities, such as proliferation, migration, antiapoptosis, mitogenesis, transformation, and angiogenesis, in many types of solid tumors, including hepatocellular carcinomas [10, 11]. However, the diagnostic value of serum MDK for hepatocellular carcinomas, particularly for those at the early stage, has not yet been investigated in Egypt.

In this study we aimed to investigate the diagnostic utility of midkine in patients with newly diagnosed hepatocellular carcinoma (HCC).

## 2. Subjects and Methods

The study protocol conformed to medical research ethical guidelines. After approval of the Research and Ethics Committee of Ain Shams University, Cairo, Egypt, in accordance with local research governance requirements, the study was carried out at HCC Clinic, Clinical Pathology, and Tropical Medicine Departments, Ain Shams University Hospitals during the interval between September 2013 and April 2014. An informed consent was taken from each participant before enrollment in the study.

This study was conducted on 100 subjects who were divided into 3 groups; group 1 included 40 patients with newly diagnosed HCC, group 2 included 30 patients with liver cirrhosis (LC), and group 3 included age- and sex-matched apparently healthy subjects serving as a control group.

Liver cirrhosis was documented by clinical assessment, laboratory findings, and evidence of liver cirrhosis upon abdominal ultrasound. The diagnosis of HCC was confirmed according to American Association for the Study of Liver Diseases (AASLD) guidelines in 2011 [12]. Patients with previous HCC treatment and liver tumors other than HCC and those with Barcelona Clinic Liver Cancer (BCLC) stage D were excluded from the study.

The enrolled patients were subjected to full medical history taking, thorough clinical examination, and laboratory investigations including complete blood picture, complete liver and kidney profile, viral or autoimmune liver markers, serum alpha-fetoprotein by chemiluminescent immunometric technique, serum midkine by enzyme-linked immunosorbent assay (ELISA), and abdominal ultrasound. Only those in HCC group underwent further imaging in the form of abdominal triphasic spiral CT or magnetic resonance imaging as a part of HCC diagnosis. Child-Pugh classification was used to assess the severity of liver disease in patients with HCC and those with LC.

Serum MDK was measured in all enrolled subjects using ELISA kit supplied by Glory Science (Glory Science Co., 2400 Veterans Boulevard, Suite 16-101, Del Rio, TX 78840, USA). The assay is based on a double-antibody sandwich ELISA technique for the quantitative assay of human MDK in samples. In this technique, MDK binds to monoclonal antibody enzyme well which is precoated with human MK monoclonal antibody, making a solid phase antibody. Then MDK antibody is added and combines with Streptavidin-Horseradish Peroxidase (HRP) to form an immune complex. Following incubation, MDK is removed during a wash step and then substrates A and B are added to the wells and the color of the liquid changes into blue. The colored product is formed in proportion to the amount of MDK present in the sample. The reaction is terminated by addition of sulphuric acid. The concentration of MDK in the samples is then determined by comparing the (optical density) OD of the samples to the standard curve and values were reported as ng/mL.

Statistical analyses were conducted using SPSS 17.0 and MedCalc software. The significance level is 0.05. Data were expressed as mean ± SD for quantitative parametric measures in addition to Median Percentiles for quantitative nonparametric measures and both number and percentage for categorized data. The quantitative variables were analyzed by Mann-Whitney *U* test (*Z*). Kruskal Wallis test is applied for statistical comparison between more than two sets of data if one or both of them have a skewed distribution. Pearson correlation test (*r*) was used to investigate the correlation between 2 quantitative variables. Chi square (*χ* ^2^) and Fisher's exact test (*F*) were used to examine the relationship between Categorical variables. The ROC was constructed to obtain the most sensitive and specific cutoff value for serum MDK in diagnosing HCC. Logistic Regression Model was used to combine information of MDK and AFP for diagnosis of HCC.

## 3. Results

In this study, a total of 100 persons were recruited and enrolled including 40 newly diagnosed HCC patients with median age of 52 years old (30 were males (75%) and 10 were females (25%)), 30 patients with liver cirrhosis with median age of 48 years old (18 males (60%) and 12 females (40%)), and 30 healthy individuals with median age of 45 years old (17 males (56.7%) and 13 females (43.3%)). Hepatitis C virus (HCV) was the underlying etiology of liver cirrhosis in 38 (95%) and 29 (96.7%) patients in HCC group and liver cirrhosis group, respectively, while only 2 (5%) and 1 patients (3.3%) in HCC group and liver cirrhosis group had chronic hepatitis B virus infection, respectively. The HCC group included 20 patients with BCLC stages 0 and A (very early and early HCC), 10 patients with BCLC stage B (intermediate HCC) and 10 patients with BCLC stage C (advanced HCC). Child A class was found in 34 patients (24 in HCC group and 10 in liver cirrhosis group), Child B in 26 patients (16 in HCC group and 10 in liver cirrhosis group), and Child C in 10 patients with liver cirrhosis group.

The median values of the MDK levels in the HCC group were much higher when compared to the LC group (0.625 versus 0.15 ng/mL; *P* < 0.001) and to the healthy control group (0.625 versus 0.125 ng/mL; *P* < 0.001). Meanwhile, however, the median values of MDK levels in the LC group were higher than that in the control group, yet not reaching significance (0.15 versus 0.125 ng/mL; *P* > 0.05) (Table 1). Regarding AFP, its median value was higher among HCC than LC group (39.3 ng/mL versus 17.5 ng/mL; *P* < 0.05) and healthy group (39.3 ng/mL versus 1 ng/mL; *P* < 0.001) (Table 1).

Patients with BCLC stage B/C had significant higher median AFP levels (300 ng/mL) when compared to those with stage 0/A (17 ng/mL) (*P* = 0.001), while no significant association was found between serum MDK and BCLC stage (0.526 ng/mL versus 0.75 ng/mL; *P* = 0.219). In the HCC group, there was no significant correlation between MDK with tumor diameter and number of tumor nodules (*r* = 0.125; *P* = 0.442 and *r* = 0.129; *P* = 0.427, resp.). Also serum levels of MDK did not show any significant correlation with serum levels of AFP in all studied patients (*r* = 0.107; *P* = 0.291), while serum levels of AFP showed significant positive correlation only with tumor size but not with tumor number in the 40 HCC patients (*r* = 0.36; *P* = 0.022 and *r* = 0.088; *P* = 0.588, resp.). In addition, on comparing the median values of serum MDK in child classes A, B, and C in CLD group patients using Kruskal Wallistest, there was no significant association found between serum MDK and child class (*K* = 5.936; *P* = 0.061).

**Figure 1:**
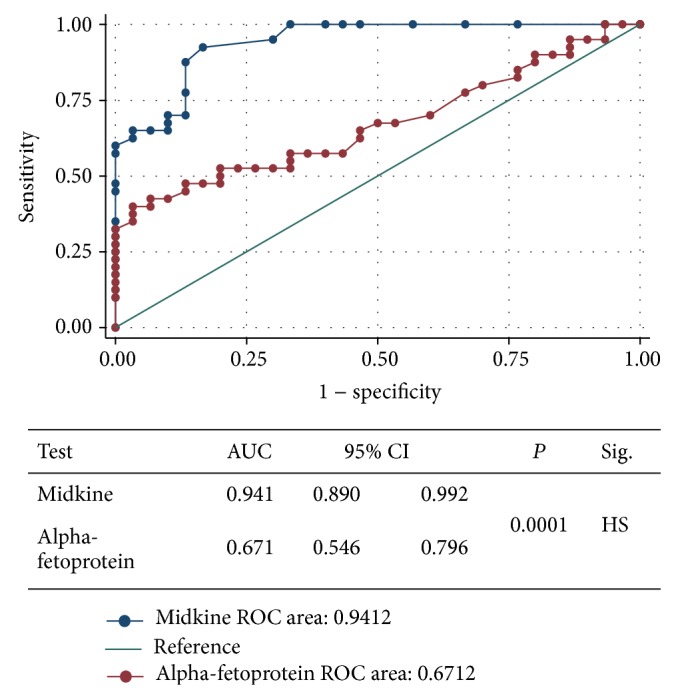
ROC curve showing comparison of the diagnostic performance of serum MDK and AFP for discriminating HCC group from chronic liver disease group.

ROC curve was performed for the best cutoff point to differentiate between HCC group and LC group using MDK and AFP. Area under the curve (AUC) for serum MDK was 0.941 (95% CI: 0.890–0.992) which was much higher when compared to that for AFP 0.671 (95% CI: 0.546–0.796) with significant statistical difference (*P* < 0.001) (Figure 1). According to the curve, the best cutoff value for MDK differentiating HCC from LC cases was 0.387 ng/mL, above which the sensitivity to discriminate HCC = 92.5% and below which the specificity to discriminate LC = 83.3% with 88.5% accuracy (true results for both), while AFP could be used to differentiate HCC from LC cases at a cutoff level of 88.5 ng/mL, with 40% sensitivity, 96.7% specificity, and 64.2% accuracy (Table 2).

To discriminate patients with HCC and those with LC, the sensitivity of MDK at cutoff value 0.387 ng/mL was found to be much significantly higher when compared to that of AFP at cutoff values 88.5, 20, and 200 ng/mL (92.5% versus 40%, 62.5%, and 25%, resp.) *P* = 0.001, while the specificity of MDK cutoff value 0.387 ng/mL was only significantly higher than that of AFP at cutoff value 20 ng/mL (83.3% versus 53.3; *P* = 0.012) (Table 3).

**Figure 2:**
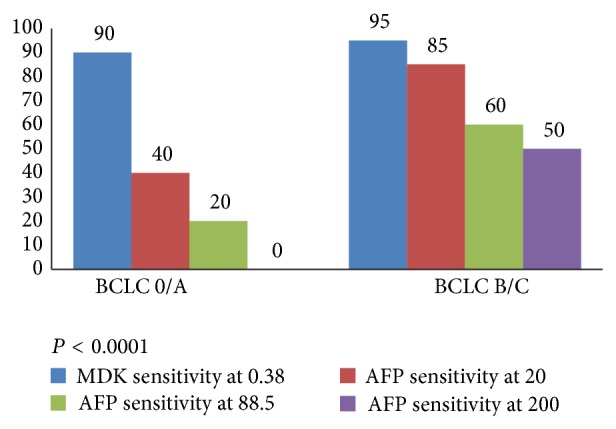
Sensitivity of MDK and AFP in diagnosis of HCC from CLD patients according to BCLC stage.

To discriminate patients with early HCC (BCLC 0/A) from those with liver cirrhosis, the sensitivity of MDK at cutoff value 0.387 ng/mL was found to be much significantly higher when compared to that of AFP at cutoff values 20, 88.5, and 200 ng/mL (90% versus 40%, 20%, and 0%, resp., *P* < 0.001) (Figure 2).

**Figure 3:**
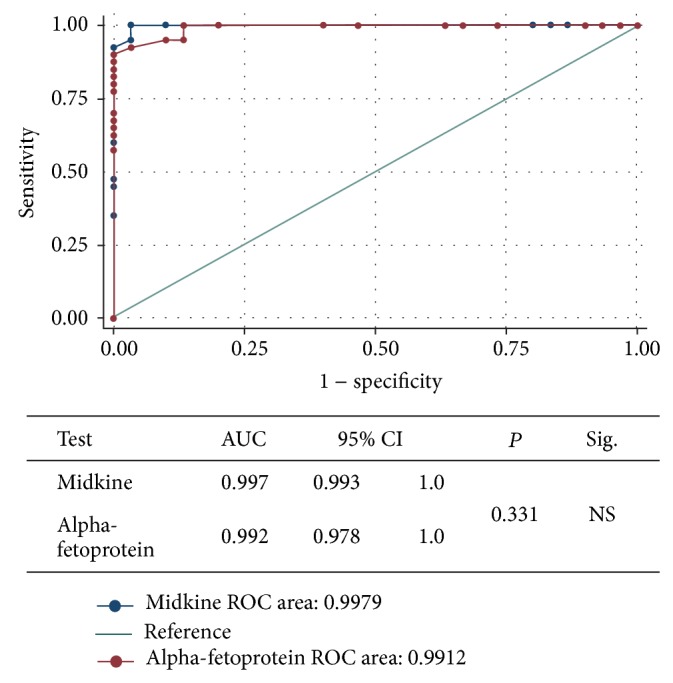
ROC curve showing comparison of the diagnostic performance of serum MDK and AFP for discriminating HCC group from healthy group.

**Figure 4:**
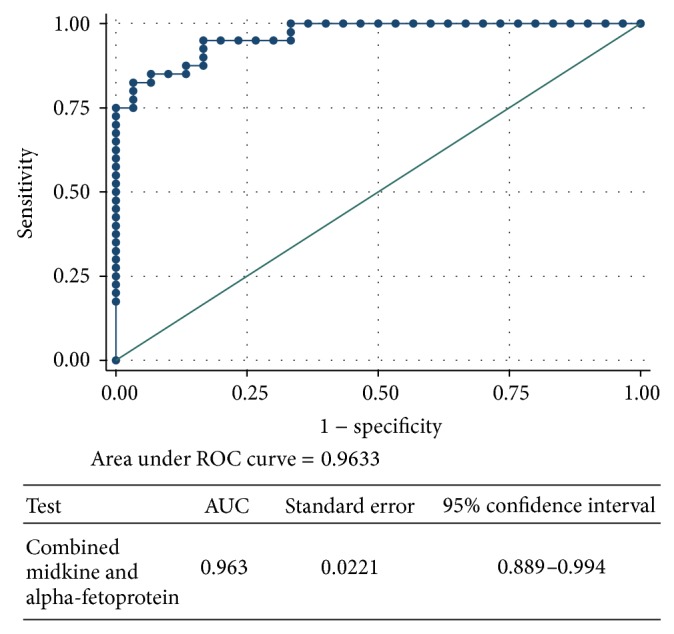
ROC curve for combined midkine and alpha-fetoprotein for discrimination between HCC and CLD.

Both of MDK and AFP had excellent diagnostic performance to differentiate HCC group from the control group with no significant statistical difference regarding their AUC (0.998 versus 0.991; *P* = 0.331), sensitivity (100% versus 92.5%), and specificity (96.7% versus 96.7%), respectively (Figure 3). The AUROC for combined serum MDK and AFP was 0.963 with *P* < 0.001 and 95% CI (0.889 to 0.994) (Figure 4).

## 4. Discussion

It has been estimated that 70% to 90% of patients with hepatocellular carcinomas have an established background of chronic liver disease or cirrhosis, the major causes of which are HBV or HCV infection [13]. In Egypt, a large study evaluated the epidemiological characteristics of HCC stated that HCV is the predominant cause of the underlying liver cirrhosis constituting about 91.32% of HCC cases while chronic HVB infection was reported in 2.51% [14]. This is very close to our results where 95% of HCC cases had chronic HCV and only 5% had chronic HBV infection.

When used as a diagnostic test, AFP levels at a value of 20 ng/mL show low specificity but fair sensitivity (60%); that is, AFP surveillance would miss 40%, whereas at higher cut-offs of 200 ng/mL the sensitivity drops to 22% with high specificity. Therefore, reducing the cutoff means that more HCCs would be identified, but at the cost of a progressive increase in the false-positive rate [15]. Consequently, AFP is an inadequate screening test [16] and is no longer assessed in surveillance programs due to the low capacity of identifying new cases not previously detected by imaging techniques [17]. These data are very similar from our findings as at cutoff values of 20 ng/mL and 200 ng/mL AFP had 62.5, 25%, sensitivity and 53.3, 100%, specificity, respectively.

In this study, we found serum MDK was significantly elevated in patients with hepatocellular carcinomas compared with liver cirrhosis patients (median: 0.625 versus 0.15 ng/mL) and the healthy controls (median: 0.625 versus 0.125 ng/mL). In addition, serum MDK was not significantly higher in the liver cirrhosis group than that in healthy group (0.15 versus 0.125 ng/mL; *P* = 1.559), in contrast to serum AFP which was significantly elevated in the liver cirrhosis group when compared to healthy group (17.5 versus 1 ng/mL; *P* = 0.0001). This means that the well-known nonspecific elevations of AFP in patients with liver cirrhosis were not significantly elicited with serum MDK increasing its specificity as a novel diagnostic marker for HCC.

Serum AFP levels were found to be significantly correlated with larger tumor size in this study (*r* = 0.36; *P* = 0.022). In addition, patients with advanced-stage hepatocellular carcinomas had significantly higher median AFP serum levels (BCLC B/C) than that of early-stage tumors (BCLC 0/A) (300 ng/mL versus 17 ng/mL, *P* = 0.0014). However, no significant correlation was found between serum MDK levels with tumor size, number or serum levels of AFP, and no significant association was found between serum MDK levels and BCLC stages.

The best cutoff values for MDK and AFP to discriminate HCC cases from those with liver cirrhosis were 0.387 and 88.5 ng/mL, respectively, with sensitivities (92.5 versus 40%), specificities (83.3 versus 96.7%), and accuracies (88.5 versus 68.2%), respectively. Then through the analysis of the ROC, the AUC of MDK (0.941) was found to be much larger than that of serum AFP (0.671; *P* < 0.001) with high significant statistical difference. This means that the overall diagnostic performance of MDK for HCC diagnosis is much better than that of AFP.

On comparing the sensitivities and specificities of MDK at the cutoff 0.387 ng/mL to those of AFP at different cutoff values (20, 88.5, and 200 ng/mL), we found that the sensitivities of MDK were significantly higher than those of AFP at all values (92.5 versus 62.5, 40, and 25%), respectively, with similar specificities to AFP at cutoffs 88.5 and 200 ng/mL, while MDK had significantly higher specificity than that of AFP only at the value of 20 ng/mL (83.3 versus 53.3).

Fifteen out of 40 HCC patients (37%) had low AFP (<20 ng); from those 15 patients, only 1 patient had MDK <0.387 ng/mL; this shows that MDK had an outstanding performance for distinguishing hepatocellular carcinomas from liver cirrhosis even in patients with AFP <20 with sensitivity reaching 93.3%. No one in the healthy control group exceeded MDK cutoff level 0.38 ng/mL and only 5 patients (16.7%) from liver cirrhosis group exceeded this threshold. On the other hand, 46.7% (14 of 30) of these patients were above the cutoff value of AFP (20 ng/mL). These indicate that MDK is a novel marker and superior to AFP with a lower false-positive rate in diagnosing and differentiating hepatocellular carcinomas from liver cirrhosis.

In terms of early detection and diagnosis of HCC, MDK at cutoff value 0.387 ng/mL showed a superior diagnostic performance to differentiate early stage HCC from patients with liver cirrhosis when compared to AFP at different cutoff values 20, 88.5, and 200 ng/mL (BCLC 0/A; sensitivity, 90% versus 40, 20, and 0%, resp.) (*P* < 0.001).

The AUC of combined MDK and AFP for discrimination between HCC and liver cirrhosis patients was larger than that MDK alone (0.963 versus 0.941) but the difference did not reach a significant level. Thus, combination of MDK and APF may be a promising strategy for early diagnosis of hepatocellular carcinomas in the future.

Our results are close to those reported by Zhu et al. [18]. That study involved three independent cohorts with a total of 933 participants including 388 HCC cases and 545 different controls enrolled from different medical centers. Results showed that MDK levels were significantly elevated in HCC tissues as well as serum samples; serum MDK at the cutoff value of 0.654 ng/mL for HCC diagnosis showed an obviously higher sensitivity compared with AFP (86.9% versus 51.9%) with similar specificities (83.9% versus 86.3%); even in very early-stage HCC, the sensitivity of MDK was significant higher than AFP (80% versus 40%); in those AFP-negative HCC cases, the sensitivity could reach as high as 89.2%; and serum MDK level was significantly decreased in HCC patients after curative resection and reelevated when tumor relapsed [18]. But the value of MDK in detection of HCV-related early HCC was not analyzed in that study, which was investigated in the current study.

In conclusion, serum MDK may serve as a novel diagnostic tumor marker for the detection of hepatocellular carcinomas, particularly in patients with AFP <20 ng/mL and/or at an early stage. Further studies with larger population are needed to justify its implementation in clinical practice.

## Figures and Tables

**Table 1:** Comparison between each of the two studied groups regarding serums AFP and MDK.

Parameters	MDK (ng/mL)		Significance		AFP (ng/mL)		Significance	
	Median	1st and 3rd quartile	*Z*	*P*	Median	1st and 3rd quartile	*Z*	*P*
HCC (*n* = 40) versus LC (*n* = 30)	0.625 0.15	0.45–0.96 0.12–0.23	6.278	0 (HS)	39.3 17.5	13.5–225.5 10–36	2.223	0.026 (S)

HCC (*n* = 40) versus healthy (30)	0.625 0.125	0.45–0.96 0.1–0.15	7.085	0 (HS)	39.3 1	13.5–225.5 0.5–2	6.914	0 (HS)

LC (*n* = 30) versus healthy (30)	0.15 0.125	0.12–0.23 0.1–0.15	1.559	0.118 (NS)	17.5 1	10–36 0.5–2	6.290	0 (HS)

MDK: midkine, AFP: alpha fetoprotein, HCC: hepatocellular carcinoma, and LC: liver cirrhosis.

**Table 2:** The diagnostic performance of the best cutoff values of AFP and MDK for discriminating HCC group from LC group.

	Cutoff value	AUC (95% CI)	Sensitivity (%)	Specificity (%)	PPV (%)	NPV (%)	Accuracy (%)	*P*
MDK	0.387	0.941 (0.890–0.992)	92.5	83.3	88	89.2	88.5	0.001

AFP	88.5	0.671 (0.546–0.796)	40	96.7	94.1	54.7	64.2	0.015

MDK: midkine; AFP: alpha-fetoprotein; AUC; area under the curve; CI: confidence interval; PPV: positive predictive value; NPV: negative predictive value.

**Table 3:** Comparison between MDK at cutoff value 0.387 ng/mL and AFP at cutoff values 88.5, 20, and 200 ng/mL regarding sensitivity and specificity in diagnosis of HCC.

	Cutoff	Sensitivity		Specificity	
		%	*P*	%	*P*
Midkine versus AFP	0.387 88.5	92.5 40	0.001^*^ HS	83.3 **96.7**	0.195^**^ NS

Midkine versus AFP	0.387 20	92.5 **62.5**	0.001^*^ HS	83.3 **53.3%**	0.012^*^ S

Midkine versus AFP	0.387 200	92.5 **25.0%**	0.001^*^ HS	83.3 100	0.052^**^ NS

MDK: midkine; AFP: alpha-fetoprotein.

^*^Chi-square test.

^**^Fisher's exact test.

## References

[1] Parkin D. M., Bray F., Ferlay J., Pisani P. (2005). Global cancer statistics, 2002. *CA: A Cancer Journal for Clinicians*.

[2] http://www.nci.cu.edu.eg/.

[3] European Association for the Study of the Liver (2012). EASL-EORTC clinical practice guidlines: managment of hepatocellular carcinoma. *Journal of Hepatology*.

[4] di Bisceglie A. M., Sterling R. K., Chung R. T. (2005). Serum alpha-fetoprotein levels in patients with advanced hepatitis C: results from the HALT-C Trial. *Journal of Hepatology*.

[5] Yamashita T., Forgues M., Wang W. (2008). EpCAM and alpha-fetoprotein expression defines novel prognostic subtypes of hepatocellular carcinoma. *Cancer Research*.

[6] Villanueva A., Minguez B., Forner A., Reig M., Llovet J. M. (2010). Hepatocellular carcinoma: novel molecular approaches for diagnosis, prognosis, and therapy. *Annual Review of Medicine*.

[7] Koike Y., Shiratori Y., Sato S. (2001). Des-*γ*-carboxy prothrombin as a useful predisposing factor for the development of portal venous invasion in patients with hepatocellular carcinoma: a prospective analysis of 227 patients. *Cancer*.

[8] Sterling R. K., Jeffers L., Gordon F. (2007). Clinical utility of AFP-L3% measurement in North American patients with HCV-related cirrhosis. *The American Journal of Gastroenterology*.

[9] Ibusuki M., Fujimori H., Yamamoto Y. (2009). Midkine in plasma as a novel breast cancer marker. *Cancer Science*.

[10] Kato M., Shinozawa T., Kato S., Awaya A., Terada T. (2000). Increased midkine expression in hepatocellular carcinoma. *Archives of Pathology and Laboratory Medicine*.

[11] Muramatsu T. (2002). Midkine and pleiotrophin: two related proteins involved in development, survival, inflammation and tumorigenesis. *Journal of Biochemistry (Tokyo)*.

[12] Bruix J., Sherman M. (2011). Management of hepatocellular carcinoma: an update. *Hepatology*.

[13] El-Serag H. B., Rudolph K. L. (2007). Hepatocellular carcinoma: epidemiology and molecular carcinogenesis. *Gastroenterology*.

[14] Shaker M. K., Abdella H. M., Khalifa M. O., ElDorry A. K. (2013). Epidemiological characteristics of hepatocellular carcinoma in Egypt: a retrospective analysis of 1313 cases. *Liver International*.

[15] Trevisani F., D'Intino P. E., Morselli-Labate A. M. (2001). Serum alpha-fetoprotein for diagnosis of hepatocellular carcinoma in patients with chronic liver disease: influence of HBsAg and anti-HCV status. *Journal of Hepatology*.

[16] Sherman M. (2001). Alphafetoprotein: an obituary. *Journal of Hepatology*.

[17] Llovet J. M., Bruix J. (2008). Novel advancements in the management of hepatocellular carcinoma in 2008. *Journal of Hepatology*.

[18] Zhu W.-W., Guo J.-J., Guo L. (2013). Evaluation of midkine as a diagnostic serum biomarker in hepatocellular carcinoma. *Clinical Cancer Research*.

